# Ventilatory and ECMO treatment of H1N1-induced severe respiratory failure: results of an Italian referral ECMO center

**DOI:** 10.1186/1471-2466-11-2

**Published:** 2011-01-11

**Authors:** Giovanni Cianchi, Manuela Bonizzoli, Andrea Pasquini, Massimo Bonacchi, Giovanni Zagli, Marco Ciapetti, Guido Sani, Stefano Batacchi, Simona Biondi, Pasquale Bernardo, Chiara Lazzeri, Valtere Giovannini, Alberta Azzi, Rosanna Abbate, Gianfranco Gensini, Adriano Peris

**Affiliations:** 1 Anesthesia and Intensive Care Unit of Emergency Department, Careggi Teaching Hospital, Largo Brambilla 3, 50139, Florence, Italy; 2 Heart and Vessels Department, Careggi Teaching Hospital, Largo Brambilla 3, 50139, Florence, Italy; 3 Post graduated school of Anesthesia and Intensive Care, University of Florence, Largo Brambilla 3, 50139, Florence, Italy; 4 Regional Health System, Viale Pieraccini 28, 50134, Florence, Italy; 5 Department of Public Health, University of Firenze, Viale Morgagni 48, 50139, Florence, Italy; 6 Department of Critical Care Medicine and Surgery, Careggi Teaching Hospital, Largo Brambilla 3, 50139, Florence, Italy

## Abstract

**Background:**

Since the first outbreak of a respiratory illness caused by H1N1 virus in Mexico, several reports have described the need of intensive care or extracorporeal membrane oxygenation (ECMO) assistance in young and often healthy patients. Here we describe our experience in H1N1-induced ARDS using both ventilation strategy and ECMO assistance.

**Methods:**

Following Italian Ministry of Health instructions, an Emergency Service was established at the Careggi Teaching Hospital (Florence, Italy) for the novel pandemic influenza. From Sept 09 to Jan 10, all patients admitted to our Intensive Care Unit (ICU) of the Emergency Department with ARDS due to H1N1 infection were studied. All ECMO treatments were veno-venous. H1N1 infection was confirmed by PCR assayed on pharyngeal swab, subglottic aspiration and bronchoalveolar lavage. Lung pathology was evaluated daily by lung ultrasound (LUS) examination.

**Results:**

A total of 12 patients were studied: 7 underwent ECMO treatment, and 5 responded to protective mechanical ventilation. Two patients had co-infection by Legionella Pneumophila. One woman was pregnant. In our series, PCR from bronchoalveolar lavage had a 100% sensitivity compared to 75% from pharyngeal swab samples. The routine use of LUS limited the number of chest X-ray examinations and decreased transportation to radiology for CT-scan, increasing patient safety and avoiding the transitory disconnection from ventilator. No major complications occurred during ECMO treatments. In three cases, bleeding from vascular access sites due to heparin infusion required blood transfusions. Overall mortality rate was 8.3%.

**Conclusions:**

In our experience, early ECMO assistance resulted safe and feasible, considering the life threatening condition, in H1N1-induced ARDS. Lung ultrasound is an effective mean for daily assessment of ARDS patients.

## Background

Since the first outbreak of a respiratory illness caused by Influenza A (H1N1) virus in Mexico [1], several reports have described the need of intensive care [2-4] or extracorporeal membrane oxygenation (ECMO) assistance [5] in young and often healthy patients.

Beginning August 2009, the Italian Ministry of Health and the Tuscany Ministry of Health issued instructions to identify and establish referral centers able to care for the more severely ill influenza patients. Therefore, several referral centers were identified throughout the national territory among the hospitals already experienced in extracorporeal respiratory support techniques. The referral ECMO centres, in addition to being capable of guaranteeing the most advanced treatment in influenza related respiratory failure, were also entrusted with providing support to the nearby hospitals and assuring safe transportation.

In the present investigation we report our experience, as an ECMO referral center, in H1N1-induced acute respiratory distress syndrome (ARDS) and we present the critical care service planning in response to the H1N1 pandemic.

## Methods

Following the instructions of the Italian Ministry of Health and Tuscany Regional Ministry of Health, an Emergency Medical Service was established in the Careggi Hospital in Florence Italy for the novel pandemic influenza.

The Careggi Hospital ECMO Team is composed of: an intensivist, a cardiac surgeon, a cardiologist, a nurse, and a perfusionist. All of the members of the team are properly trained in ECMO treatment. An ambulance and a car are equipped with an ECMO circuit, a transport ventilator and all of the materials needed to initiate extracorporeal support in the peripheral hospitals, and permit safe transportation while on extracorporeal circulation to our referral hospital.

The requirement of ECMO was decided based on the Italian Ministry of Health criteria (Table 1).

**Table 1:** Italian Ministry of Health criteria to discuss the need of ECMO.

**Acute respiratory failure with one of the following condition**:
1. SaO_2 _< 85% for at least 1 hour
2. Oxygenation Index >25 for at least 6 hours after ventilation optimisation
3. PaO_2_/FiO_2 _< 100 with PEEP ≥ 10cmH2O for at least 6 hours after ventilation's optimization
4. Hypercapnia with pH < 7.25
5. SvO_2 _< 65% with hematocrit >30 and under vasoactive drugs infusion

The parameter are referred to a condition of lung protective ventilation (tidal volume:4-6 ml/Kg of predicted body weight; plateau pressure ≤ 30 cmH_2_0; PEEP >lower inflection point of the curve pressure-volume).

PEEP: positive end-expiratory pressure; PaO_2_: arterial oxygen partial pressure; FiO_2_: inspired oxygen fraction; RR: respiratory rate; SaO_2_: peripheral oxygen saturation; SvO_2_: central venous oxygen saturation.

Oxygenation Index: Mean airway pressure (cmH_2_O) * FiO_2 _* 100/PaO_2_;

From September 2009 to January 2010, all patients admitted to our ICU with severe respiratory failure due to H1N1 infection were included in this study. Patient demographics and clinical characteristics were collected from institutional ICU database (FileMaker Pro, FileMaker, Inc, USA), from Italian Group for the Evaluation of Interventions in Intensive Care Medicine database (GiViTI Margherita Project, Istituto Mario Negri, Bergamo, Italy) and from ECMO national network database. Discrete variables are expressed as counts and percentages, whereas continuous variables are reported as medians with 25th to 75th interquartile range (IQR). The Internal Review Board approved this retrospective study and informed consent for data publication was obtained from the patients or relatives.

### Ventilation strategy

Pressure volume curves were calculated with ventilator's built in application (Draeger Evita XL, Draeger Medical AG, Lubeck Germany) starting from a PEEP level of 5 cm H_2_O, with a pressure limit of 40. Ventilation parameters were set on the basis of this calculation, with a PEEP of 2 cmH_2_O above the lower inflection point of the pressure-volume curve, and a peek pressure below the upper inflection point. In all cases, pressure plateau was limited to 30 cmH_2_O and the tidal volume was kept below 6 ml/Kg [6]. Recruitment manoeuvres (40 sec at 40 cmH_2_O) were performed twice a day, if needed, to improve pulmonary gas exchange.

### ECMO

All ECMO treatments were veno-venous (Maquet Rotaflow Centrifugal Pumps with Quadrox-D oxygenators, Maquet, Rastatt, Germany) and biocoated circuits were used.

Two types of cannulation were used. Initially a venous withdrawal cannula was inserted via femoral vein (Edwards Lifesciences Femoral Venous Cannula 22-24 Fr., Edwards Lifescience, Irvine, CA-USA or Maquet HLS Venous Cannula 21-25 Fr., Maquet Cardiopulmonary AG, Hirrlingen-Germany) and an infusion cannula in the right jugular vein (Edwards Lifesciences Fem-Flex II Cannula 20 Fr., Edwards Lifescience, Irvine, CA-USA or Maquet HLS Arterial Cannula 23 Fr., Maquet Cardiopulmonary AG, Hirrlingen-Germany) were adopted. During the period study Avalon double lumen cannulas (Avalon Elite Bi-Caval Dual Lumen Catheter 27-31 Fr., Avalon Laboratories, Rancho Dominguez, CA - USA) become available and were inserted through internal right jugular vein in 2 patients.

Cannulation was conducted percutaneously with Seldinger technique in all cases, and cannulas position was confirmed by transesophageal echocardiography.

Heparin infusion during extracorporeal lung assistance was monitored every two hours by bedside aPTT measurement (Hemochron Jr. Signature plus, ITC Europe, Milan, IT), which was maintained between 50 and 80 seconds. In case of renal replacement therapy requirement in ECMO patients, a continuous veno-venous hemodiafiltration circuit was assembled on the ECMO circuit (aspiration on pre-pump line, restitution on pre-oxygenation line).

ECMO patients were ventilated with protective parameters, and respiratory rate and ECMO flow were adjusted to achieve normocarbia and oxygen saturation above 92%.

### Infection control

H1N1 infection was confirmed by real-time reverse transcriptase-polymerase-chain-reaction (RT-PCR) assayed on pharyngeal swab, subglottic aspiration and bronchoalveolar lavage in accordance with published guidelines [7]. Bronchoalveolar specimens were obtained with a mini-invasive system (Kimberly-Clark BAL Cath, Kimberly-Klark N.V. Zaventem - Belgium), or by bronchoscopy.

Patients were isolated in negative pressure atmosphere rooms, and staff wore full protective garments (including FFP3 respirators, 3 M Italia SpA, Segrate, Italy), until 2 consecutive tests were confirmed negative. During the study period only one case of suspected transmission of influenza to a nurse occurred.

Antiviral therapy consisted in oral oseltamivir (75 mg twice daily) and inhaled zanamivir (10 mg twice daily).

Blood and urinary cultures, tracheal aspirate, and pharyngeal swab were obtained upon patient admission. Empiric antimicrobial regimen at ICU admission was initiated with levofloxacin and amoxicillin/clavulanate; eventually specific antimicrobial therapy was varied or ended on the basis of microbiological results.

Steroids were administered at low dosage (20 mg metilprednisolon twice per day) to prevent lung fibrosis. Diuretics were administered at different dosages, depending on clinical judgment and the patient's renal function.

### Lung ultrasound examination

Lung ultra sound (LUS) examinations were daily performed by the attending physician, with a multi-frequency convex probe (3.5-5 MHz, Mylab TM 30CV, ESAOTE, Genova, IT). With the patient in semirecumbent position, lateral and anterior views were obtained from base to apex of the chest. Posterior axillary line was followed during lateral transversal examinations. Chest quadrants defined by the intercostal spaces and the parasternal, mid-clavicular, and anterior axillary lines were scanned on the anterior chest wall [8]. The occurrence and extension of parenchymal consolidations, alveolar interstitial syndrome (measured by the number of B-lines), and morphology of pleural line were evaluated [9-11]. Pleural effusions were estimated by using Balik's formula [12,13]. In order to ensure a uniform record, and allow to follow the evolution of the findings over time, all exams were recorded in an electronic form, in which the description of the main LUS features was predetermined [14].

## Results

### Overall patients

During the study period, 12 patients requiring invasive ventilation treatment and/or ECMO were admitted or transferred to our ICU. Baseline and clinical characteristics of patients admitted for H1N1-induced severe respiratory failure are summarized in Table 2.

**Table 2:** baseline and clinical characteristics of H1N1-pneumonia patients.

Number	12
**Male sex, N (%)**	8 (66 .7%)

**Age (years)**	44.5 (36.8-48.8)

**BMI**	27 (23.8-31)

**SAPS II**	36 (27.75-44.75)

**Patients with comorbidities, N (%)**	5 (41 .7%)

**Patients with proved coinfection, N (%)**	2 (16 .7%)

**Days from onset to ICU admission**	7 (6-8.25)

**§ PaO**_**2**_**(mmHg)/FiO**_**2**_	92 (53.5-119.5)

**§ PaCO**_**2 **_**(mmHg)**	65 (39.9-83)

**§ pH**	7.37 (7.32-7.50)

**§ Respiratory rate (N/min)**	8.62 (6.87-11.3)

§ White cells count (N*1000/ml)	8.620 (6.870-11.300)

§ Platelets count (N*1000/ml)	158.5 (102-217.3)

**§ Lactate dehydrogenase (U/l)**	617 (391.2-919.8)

**§ Creatine kinase (U/l)**	611 (402.5-893.8)

**§ Aspartate aminotransferase (U/l)**	53.5 (39.8-121.3)

**§ Alanine aminotransferase (U/l)**	37.5 (27.3-43.5)

**§ C-reactive protein (mg/dl)**	80.5 (35.9-132.4)

**§ Serum creatinine (mg/dl)**	0.87 (0.69-1.26)

**§ Procalcitonin (ng/ml)**	3.2 (1.1-4.3)

**Chest radiographs **(mean per patient)	7.3

**Chest CT-scan **(mean per patient)	1.25

**Tracheostomy, N (%)**	5 (41 .7%)

**ECMO, N (%)**	7 (58 .3%)

**CVVH, N (%)**	2 (16 .7%)

**Duration of mechanical ventilation (days)**	13.5 (10.8-21.5)

**ICU lenght of stay (days)**	16.5 (10.5-25.5)

**Mortality, N (%)**	1 (8 .3%)

Data are referred to first assessment at ICU admission. Continuous data are represented as medians with 25th to 75th interquartile range (IQR). Percent data are referred to the total population of each group.

BMI: body mass index; CVVH: continuous veno-venous hemofiltration; ECMO: extracorporeal membrane oxygenation; SAPS: simplified acute physiology score.

§ values at ICU admission

The median time between initial, non specific, symptoms and respiratory failure was 7 days (IQR 6-8.25), and severe hypoxia, unresponsiveness to non-invasive ventilation, was the main clinical feature. Our patients were young, median age 44.5 years, none of them older than 58 years, and eight (80%) younger than 50. Two patients were severely obese (BMI > 40), one woman was pregnant (18 weeks), two patients had a history of chronic obstructive respiratory disease (COPD), and one had diabetes. Two patients had Legionella Pneumophila coinfection at admission, and one young patient (16 years old) with suspect viral myocarditis and heart failure. At admission the patients, with the exception of the two coinfected, presented low leukocyte and platelet count and low plasma procalcitonin levels, significant levels of lactate dehydrogenase (LDH), creatine kinase (CK), and C-reactive protein (Table 2). Median duration of mechanical ventilation (days) was 11.5 (IQR 9.8-16.3) and median ICU length of stay (days) was 14 (IQR 12-16.5). The pregnant woman continued the pregnancy without significant complications.

In ICU infection rate was low with two ventilator associated pneumonia and two asymptomatic positive blood cultures in two ECMO patients. One ECMO patient died due to a systemic secondary infection by Aspergillus: this patient was the only non-surviving patient (overall mortality rate 8.3%).

### H1N1 infection monitoring and therapy

**Figure 1:**
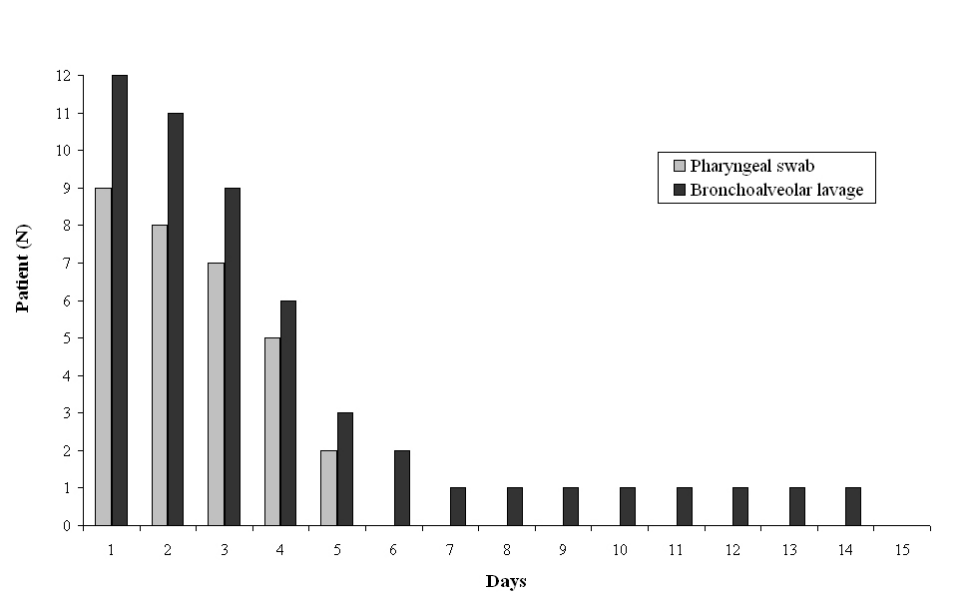
Time course of RT-PCR results on pharyngeal swab and bronchoalveolar lavage.

RT-PCRs from bronchoalveolar lavage samples were positive in all patients included in this study. On the contrary, RT-PCR dosed on pharyngeal swab resulted positive in less than 70% of patients at ICU admission, and in 90% of patients in the second day (Figure 1). Also efficacy of antiviral therapy was reliably followed through RT-PCR from bronchoalveolar samples, since analysis on pharyngeal swabs became negative quite early. Finally, no RT-PCR significant for H1N1 infection from subglottic aspirate sample was found.

In one patient, intravenous administration of zanamivir was needed, since the patient remained positive to viral infection after two weeks of therapy. Intravenous formulation of zanamivir is still subjected to pre-phase 4 clinical trial investigation, even if some reports on its safety profile are already available in literature. Therefore, local Ethical Committee approval was requested and the manufacturer provided the drug for use. Zanamivir was administered intravenously for five days (600 mg twice daily), as indicated by the producer. The patient's respiratory function improved and RT-PCR became negative after the third day. No adverse reaction was noted.

### Lung ultrasound (LUS) examination in H1N1-induced ARDS

A total of 156 LUS have been performed. During every LUS, the following parameters were considered: pleural line aspect and motility, presence of consolidations, occurrence and severity of Alveolar Interstitial Syndrome (based on the number of B-lines), presence of pleural effusion and occurrence of pneumothorax.

Pleural thickness was described in 100% of cases and mostly bilaterally. Lung base was always involved. Lung gliding was present in 70% of LUS, even if decreased (20%). Pathological Lung Pulse was found in 20% of LUS, often in proximity to large parenchyma consolidations. Pleural effusion occurred in 7 patients. Two spontaneous pneumothorax have been detected with LUS during ICU treatment.

Alveolar Interstitial Syndrome was present in all ultrasound examinations, with the presence of normal lung pattern (spared areas). In 90% of cases, B-Lines were described as moderate/many. At lung recovery, residual B-Lines patterns were found mostly at both bases. White lung feature occurred in about 15% of LUS performed, mostly in the anterior and lateral scans. White lung was never uniformly distributed, but it was alternated to spared areas, or areas with a limited number of B-Lines.

Consolidations were found in 100% of cases. Most of them were multiple (65%), and lung bases were always involved. Contiguous subpleural consolidations were also present, increasing the pleural thickness laterally, mostly at the base and the apical part. Aerial bronchograms were always found within the consolidation pattern.

The routine use of LUS limited the number of conventional radiology examinations (Table 3). In ECMO patients group, the higher number of chest X-ray examinations was needed to verify the correct cannulae positioning. In both groups, bedside LUS limited the transportation to the CT-scan room, increasing patient safety and avoiding the transitory disconnection of the patient from the ventilator.

**Table 3:** differences between ECMO and ventilated patients.

	ECMO patients	Non-ECMO patients
**Number (%)**	7	5

**Male sex, N (%)**	6 (85 .7%)	2 (40%)

**Age (years)**	45 (37-46.5)	42 (39-51)

**BMI**	27 (23.5-36.5)	27 (25-29)

**SAPS II**	44 (39-50.5)	28 (27-28)

**Patients with comorbidities, N (%)**	5 (71 .4%)	2 (40%)

**Patients with proved coinfection, N (%)**	2 (28 .6%)	0

**Days from onset to ICU admission**	6 (6-7.5)	7 (7-9)

**§ PaO**_**2**_**(mmHg)/FiO**_**2**_	48.7 (46.8-58)	116 (107-141)

**§ PaCO**_**2 **_**(mmHg)**	81.6 (60.6-88)	57 (55.8-63)

**§ pH**	7.25 (7.20-7.30)	7.33 (7.28-7.34)

**§ Respiratory rate (N/min)**	10 (8.5-15)	20 (18-22)

**§ PEEP (cmH**_**2**_**O)**	14 (11-15)	14 (11-15)

**§ Static compliance(ml/cmH**_**2**_**O)**	34 (30-38)	32 (23-38)

§ White cells count (N*1000/ml)	11 (9.1-13.3)	7.03 (6.7-8.6)

§ Platelets count (N*1000/ml)	151 (115.5-218.5)	158.5 (125.3-182.5)

**§ Lactate dehydrogenase (U/l)**	445 (372.5-920.5)	627 (607-632)

**§ Creatine kinase (U/l)**	477 (189-890.3)	435 (281.5-695)

**§ Aspartate aminotransferase (U/l)**	54 (42-121.5)	53 (42-78)

**§ Alanine aminotransferase (U/l)**	38 (25.5-41)	37 (29-45)

**§ C-reactive protein (mg/dl)**	83.5 (77.5-315.7)	127.5 (96.3-158.8)

**§ Serum creatinine (mg/dl)**	1.02 (0.79-1.72)	0.81 (0.55-0.85)

**§ Procalcitonin (ng/ml)**	4.3 (2.7-29.4)	1.9 (0.2-2.6)

**CVVH, N (%)**	2 (28 .6%)	0

**Chest radiographs (mean per patient)**	9.6	4.2

**Chest CT-scan (mean per patient)**	1.6	0.8

**Duration of mechanical ventilation (days)**	19 (12-36)	9 (5-11)

**ICU lenght of stay (days)**	23 (17.5-38)	11 (8-14)

**Packed Red Blood Cells Units, N**	4 (1-6)	1 (0-3)

**Mortality, N (%)**	1 (14.3%)	0 (0%)

Data on airway pressures and respiratory rate are referred after intubation and recruitment. Continuous data are represented as medians with 25th to 75th interquartile range (IQR). Percent data are referred to the total population of each group.

BMI: body mass index; CVVH: continuous veno-venous hemofiltration; ECMO: extracorporeal membrane oxygenation; SAPS: simplified acute physiology score.

§ Values at ICU admission

### ECMO patients

ECMO was needed in 7 patients (Table 3). In 4 cases, the ECMO Team was alerted and extracorporeal oxygenation was implanted directly at peripheral ICUs. No major transportation related problems were faced, even in the case of a long distance journey (400 Km). Median duration of ECMO support was 8 days (IQR 6-16.5), with a median duration of mechanical ventilation (days) of 19 (IQR 12-36).

Main clinical features and ventilatory and ECMO parameters of patients treated with ECMO are presented in Table 4. Bleeding was the most important complication. In three cases, bleeding from vascular access sites due to heparin infusion required blood transfusions. Three patients presented prolonged oropharyngeal bleeding and transfusions were required. Among them, one needed electrical coagulation of a palatine injury, probably related to nursing manoeuvres. Two patients presented severe intra-bronchial bleeding, and several flexible bronchoscopy examinations and clot suctions were required. In one of these patients, bleeding from the lower airways during the weaning phase from ECMO, and ECMO removal has been hastened.

**Table 4:** main ventilation and ECMO data of patients treated with ECMO

			Pre-ECMO parameters								In-ECMO parameters (Day 1)						
							
Patient	Age (years)	Comorbidity	Peak Pressure	PEEP	tV	RR	**PaO**_2_*	**CO**_**2**_	pH	Ventilation days before ECMO	Peak Pressure	PEEP	tV	RR	Pump Flow	Days on ECMO	Ventilation days after ECMO
**1**	15	Asthma	30	14	350	14	50	52	7.18	1	18	10	300	10	5.2	6	3

**2**	58	-	30	14	400	16	66	76	7.14	2	22	14	300	7	5.0	6	5

**3**^**§**^	44	Obesity	33	15	240	20	60	89	7.27	2	28	15	200	9	6.1	15^	12

**4**^**§**^	48	Cigarette smoke	40	10	700	18	58	54	7.29	1	24	14	270	7	5.9	8	10^

**5**^**§**^	30	Asthma, obesity, cigarette smoke	35	15	700	22	48	55	7,22	2	24	14	400	6	5.3	6	3

**6**^**§**^	45	Psychiatric disorder, emphysema bulla	45	18	250	20	54	73	7.22	6	28	17	160	8	5.7	37^#^^	-

**7**	45	-	35	15	410	32	73	84	7.17	7	25	15	300	20	5.0	18	21^

PEEP: positive end expiratory pressure; RR: respiratory rate; tV: tidal volume.

* Measured with FiO2 = 1

§ Patient referred from another hospital

# Dead on ECMO

^ patient tracheostomized during ECMO

Table 3 summarizes the main differences between patients who underwent to ECMO treatment and patients only ventilated. Despite the small sample, ECMO patients clearly showed a higher critical illness score (SAPS II), and worst pulmonary gas exchange compared to patients who did not required extracorporeal lung assistance. Coinfection and comorbidities at admission were present only in ECMO patients.

## Discussion

Our study population is young, comprising mainly healthy subjects, as previously reported [1,2,15]. Risk factors are similar to other studies, such as obesity, diabetes and pregnancy.

In the present case series, bacterial infection rate at presentation was low. Previous reports showed incidence of secondary superinfection by Streptococcus Pneumoniae, Staphylococcus Aureus, Pseudomonas Aeruginosa, Acinetobacter Baumannii, Escherichia coli [1,3,2]. In our experience, we found two cases (16.7%) of co-infection with Legionella Pneumophila, which is, to the best of our knowledge, a new epidemiological data, since no other case has been reported in literature. It is questionable whether Legionella Pneumophila infection occurred before or after H1N1 pneumonia. However, it could be that H1N1 pneumonia was associated with a lower reactivity of the immune system, as suggested by the low leucocytes count reported in our sample and by other Authors [3,2,1].

One young patient presented heart failure, and viral myocarditis was suspected. The association of influenza with myocarditis is debated [16], and H1N1 related myocarditis, has rarely been reported [17]. Furthermore, in our patient prolonged pre-hospital hypoxia was present and myocardial hypoxemia damage might have been involved. The patient required inotrope/vasoactive support for several days and eventually recovered fully with normal heart function.

Our observations confirm the responsiveness of this infection to antiviral therapy. We adopted a two-modality administration, both oral and inhaled. Our choice was made in consideration of the decrease in gut motility and adsorption usually observed in critically ill patients.

The World Health Organization (WHO) has questioned the sensibility of RT-PCR analysis for H1N1 in pharyngeal swab sample, encouraging analysis on samples from the lower respiratory tract. We routinely monitor H1N1 infection on three compartments: pharyngeal swab, subglottic aspiration, and bronchoalveolar lavage. In our experience, bronchoalveolar lavage at admission was positive in all patients while pharyngeal swab resulted positive in only 75% of cases.

As shown in Figure 1, RT-PCR from pharyngeal swab at ICU admission failed to demonstrate the viral infection in 3 patients. Similarly, the time course showed that RT-PCR from pharyngeal swab resulted negative in an average time of 3 days after therapy start. Conversely RT-PCRs from bronchoalveolar lavage remained positive for a longer period and resulted more reliable for infection monitoring and assessment of the efficacy of administered therapy.

Based on our experience, RT-PCR from bronchoalveolar lavage resulted to be the most reliable method to diagnose and monitor H1N1 infection, since pharyngeal swab does not offer enough sensibility, neither for antiviral therapy initiation nor for antiviral therapy management. As subglottic aspiration resulted persistently negative, we do not recommend this sampling for diagnosis and monitoring of H1N1 infection.

Despite the severe clinical pictures, we experienced a very low mortality rate: only one patient out of 12 died (8,3%). One of the surviving patients presented a lung cavern for a past pulmonary infection, and deceased for a secondary superinfection by Aspergillus, probably already colonizing lung parenchyma before the onset of viral infection.

Our mortality rate is surprisingly low in comparison to a larger series of H1N1 patients, even when extracorporeal support technique were employed [5,18]. Our finding can be related to the small number of patients included the study and definitive comparison with larger studies could be misleading. However, despite the severity of symptoms and the rapid progression to ARDS, H1N1 respiratory failure presents a relatively benign course when adequately treated, if compared to non-H1N1 induced ARDS, reported to have a mortality rate from 37% to 43% [19-22]. Several factors may account for the favourable outcome in our series. All patients received protective ventilation. In particular, ECMO support permitted the maintenance of patients under a protective tidal volume with a respiratory rate below 12 per min, and a FiO_2 _below 60%, compared with non-ECMO patients who needed a higher respiratory rate and FiO_2 _to maintain an acceptable pulmonary gas exchange.

The availability of easily accessible tools for pulmonary mechanics evaluations on modern ventilators allowed an individualized and appropriate setting of ventilation pressure within the thresholds of so called "protective ventilation" [23]. Furthermore, early access to ECMO resource allowed the maintenance of protective ventilation even in more severe patients (Table 4). In this regard, lactate dehydrogenase is commonly considered a marker of lung damage, and in H1N1 pneumonia is reported as high [1]. In our ECMO patients, lactate dehydrogenase values presented lower levels than in non-ECMO patients (445 U/L vs 627 IU/L, respectively), suggesting that in ECMO patients the reduced need of pulmonary ventilation could reduce lung ventilatory stress and enhance healing, regardless of the more impaired lung condition.

However, it is possible that, since the technique has gained popularity and experience gathered to demonstrate its feasibility, we used ECMO also in patients who might previously have been successfully treated conventionally, and this may have influenced mortality.

Moreover, more than half of our ECMO patients needed to be land-transported from other hospitals in an advanced stage of respiratory failure. This may have further encouraged an early treatment with ECMO to ensure the safest transport.

Bleeding is commonly reported during ECMO treatment [24], and either anticoagulation or platelet and coagulation cascade activation through oxygenator and pump is involved [25].

In our population bleeding also occurred more frequently in ECMO patients, and they required more transfusions compared to non ECMO patients. Nevertheless, in our experience, bleeding from cannulas insertion site or from upper airways, despite requiring transfusion, were not life threatening, and could be managed. In only two cases did severe bleeding occur in the lower respiratory tract. Fortunately in one case it occurred during weaning from ECMO, and it ceased after extracorporeal support removal. The other patient died from pulmonary aspergillosis and the haemorrhage could be also related to parenchyma disruption caused by the fungus.

Monitoring heparin regimen is extremely important during extracorporeal circulation, and activated clotted time is commonly measured bedside. Some debate exists regarding the optimal range and the accuracy of point-of-care measuring devices [26-28]. In our protocol, we usually measured aPTT every two hours with Hemochron Jr. in order to closely monitor heparin administration in the low range of dosage.

In our clinical practice, lung recovery and response to treatment are daily assessed by LUS examination, following several recent reports which underline the reliability of LUS in the evaluation and management of chest disorders [10,29]. Despite CT-scan is the reference technique for evaluating lung lesions, it requires a transitory disconnection of the patient from the ventilator to permit the transportation radiology suite with potential risk of alveolar de-recruitment and worsening of oxygenation. Moreover, severe complications have been reported in intra-hospital transportation of critically ill patients [30,31]. As we recently reported [13], the routine use of bedside LUS has significantly reduce of the number of CT-scan and chest X-ray examinations in critical patients. The potential clinical benefit of reducing in-hospital transport for diagnostic radiology, it can be particularly relevant in patients with ECMO. In these patients, in fact, transportation requires time and a significant commitment of resources, although it was proved feasible both for in-hospital [32,33] and for inter-hospital long distance transportations [34,35].

Another advantage of LUS is the ability to evaluate the effectiveness of alveolar recruitment manoeuvres with the possibility to visualize real-time imagines of lung parenchyma re-aeration [8,10,29]. Finally, pleural effusions can be accurately diagnosed and monitored with LUS and in case of need for treatment an ultra-sound guided technique is recommended [36,13]. This option seems to be particularly appropriate ECMO patients, where bleeding for conventional chest tube placement can occur in consideration of the need of heparin infusion.

## Conclusions

The present case series comprises a small number of patients, and naturally, it cannot be considered a high grade of evidence trial. However, our experience might be helpful for intensivists challenging H1N1-induced ARDS. For H1N1 infection monitoring (or diagnosis, if patient was intubated before) bronchoalveolar lavage can be more reliable than pharyngeal swab in order of the higher sensitivity. In our clinical practice, ECMO therapy resulted safe and feasible in the context of a life threatening condition, and it might be taken into consideration as a therapeutic choice rather than a rescue solution in experienced centers.

## Key messages

• ECMO might be taken into consideration as a safe therapeutic choice rather than a rescue solution in ARDS.

• RT-PCR from bronchial lavage is more accurate than from pharyngeal swab, in H1N1 diagnosis.

• Lung ultrasonography is a safe and reliable method to follow the pathology evolution/recovery of lung.

• Lung ultrasonography can limit the need of CT-scan and chest X-ray examinations.

## List of abbreviations

ARDS: acute respiratory distress syndrome; BMI: body mass index; CVVH: continuous veno-venous hemofiltration; ECMO: extracorporeal membrane oxygenation; ICU: intensive care unit; LOS: length of stay; LUS: lung ultrasound; RT-PCR: real-time reverse transcriptase-polymerase-chain-reaction; SAPS: simplified acute physiology score.

## Competing interests

The authors declare that they have no competing interests.

## Authors' contributions

AP, MB, GC, AP, SB, MC, GS, MB, VG, GG organized the ECMO center. AP, MB, GC, GS, VG, GG designed the study. AP, MB, GC, AP, MC, SB, MB, SB reviewed the literature. SB collected data. PB and CL performed cardiologic and transesophageal assistance. MB performed ECMO invasive procedures. AA and RA performed laboratory and microbiological analysis; GC, GZ, SB, CL wrote the draft. All Authors have read, revised and approved the manuscript.

## Pre-publication history

The pre-publication history for this paper can be accessed here:

http://www.biomedcentral.com/1471-2466/11/2/prepub

## References

[B1] Perez-Padilla R de la Rosa-Zamboni D Ponce de Leon S Hernandez M Quinones-Falconi F Bautista E Ramirez-Venegas A Rojas-Serrano J Ormsby CE Corrales A Higuera A Mondragon E Cordova-Villalobos JA Pneumonia and respiratory failure from swine-origin influenza A (H1N1) in Mexico N Engl J Med 2009 361 7 680 689 10.1056/NEJMoa0904252 19564631

[B2] Rello J Rodriguez A Ibanez P Socias L Cebrian J Marques A Guerrero J Ruiz-Santana S Marquez E Del Nogal-Saez F Alvarez-Lerma F Martinez S Ferrer M Avellanas M Granada R Maravi-Poma E Albert P Sierra R Vidaur L Ortiz P Prieto Del Portillo I Galvan B Leon-Gil C HnSWG T Intensive care adult patients with severe respiratory failure caused by Influenza A (H1N1)v in Spain Crit Care 2009 13 5 R148 10.1186/cc8044 19747383 PMC2784367

[B3] Jain S Kamimoto L Bramley AM Schmitz AM Benoit SR Louie J Sugerman DE Druckenmiller JK Ritger KA Chugh R Jasuja S Deutscher M Chen S Walker JD Duchin JS Lett S Soliva S Wells EV Swerdlow D Uyeki TM Fiore AE Olsen SJ Fry AM Bridges CB Finelli L Hospitalized patients with 2009 H1N1 influenza in the United States, April-June 2009 N Engl J Med 2009 361 20 1935 1944 10.1056/NEJMoa0906695 19815859

[B4] Chowell G Bertozzi SM Colchero MA Lopez-Gatell H Alpuche-Aranda C Hernandez M Miller MA Severe respiratory disease concurrent with the circulation of H1N1 influenza N Engl J Med 2009 361 7 674 679 10.1056/NEJMoa0904023 19564633

[B5] Davies A Jones D Bailey M Beca J Bellomo R Blackwell N Forrest P Gattas D Granger E Herkes R Jackson A McGuinness S Nair P Pellegrino V Pettila V Plunkett B Pye R Torzillo P Webb S Wilson M Ziegenfuss M Extracorporeal Membrane Oxygenation for 2009 Influenza A(H1N1) Acute Respiratory Distress Syndrome Jama 2009 302 17 1888 1895 10.1001/jama.2009.1535 19822628

[B6] Brower RG Lanken PN MacIntyre N Matthay MA Morris A Ancukiewicz M Schoenfeld D Thompson BT Higher versus lower positive end-expiratory pressures in patients with the acute respiratory distress syndrome N Engl J Med 2004 351 4 327 336 10.1056/NEJMoa032193 15269312

[B7] CDC protocol of realtime RT-PCR for influenza A (H1N1) 2009 Geneva: World Health Organization

[B8] Peris A Zagli G Barbani F Tutino L Biondi S di Valvasone S Batacchi S Bonizzoli M Spina R Miniati M Pappagallo S Giovannini V Gensini GF The value of lung ultrasound monitoring in H1N1 acute respiratory distress syndrome Anaesthesia 2009 11 11 10.1111/j.1365-2044.2009.06210.x 20002364

[B9] Lichtenstein DA Lascols N Meziere G Gepner A Ultrasound diagnosis of alveolar consolidation in the critically ill Intensive Care Med 2004 30 2 276 281 10.1007/s00134-003-2075-6 14722643

[B10] Arbelot C Ferrari F Bouhemad B Rouby JJ Lung ultrasound in acute respiratory distress syndrome and acute lung injury Curr Opin Crit Care 2008 14 1 70 74 10.1097/MCC.0b013e3282f43d05 18195629

[B11] Schmidt GA ICU ultrasound. The coming boom Chest 2009 135 6 1407 1408 10.1378/chest.09-0502 19497889

[B12] Balik M Plasil P Waldauf P Pazout J Fric M Otahal M Pachl J Ultrasound estimation of volume of pleural fluid in mechanically ventilated patients Intensive Care Med 2006 32 2 318 321 10.1007/s00134-005-0024-2 16432674

[B13] Peris A Tutino L Zagli G Batacchi S Cianchi G Spina R Bonizzoli M Migliaccio L Perretta L Bartolini M Ban K Balik M The use of point-of-care bedside lung ultrasound significantly reduces the number of radiographs and computed tomography scans in critically ill patients Anesth Analg 2010 111 3 687 692 10.1213/ANE.0b013e3181e7cc42 20733164

[B14] Tutino L Cianchi G Barbani F Batacchi S Cammelli R Peris A Time needed to achieve completeness and accuracy in bedside lung ultrasound reporting in intensive care unit Scand J Trauma Resusc Emerg Med 2010 18 44 44 10.1186/1757-7241-18-44 20701810 PMC2928170

[B15] Webb SA Pettila V Seppelt I Bellomo R Bailey M Cooper DJ Cretikos M Davies AR Finfer S Harrigan PW Hart GK Howe B Iredell JR McArthur C Mitchell I Morrison S Nichol AD Paterson DL Peake S Richards B Stephens D Turner A Yung M Critical care services and 2009 H1N1 influenza in Australia and New Zealand N Engl J Med 2009 361 20 1925 1934 10.1056/NEJMoa0908481 19815860

[B16] Warren-Gash C Smeeth L Hayward AC Influenza as a trigger for acute myocardial infarction or death from cardiovascular disease: a systematic review Lancet Infect Dis 2009 9 10 601 610 10.1016/S1473-3099(09)70233-6 19778762

[B17] Weiss TW Stensaeth KH Eritsland J Myocarditis in a juvenile patient with influenza A virus infection Eur Heart J 2009 10 10 10.1093/eurheartj/ehp566 20007610

[B18] Freed DH Henzler D White CW Fowler R Zarychanski R Hutchison J Arora RC Manji RA Legare JF Drews T Veroukis S Kesselman M Guerguerian AM Kumar A Extracorporeal lung support for patients who had severe respiratory failure secondary to influenza A (H1N1) 2009 infection in Canada Can J Anaesth 2010 16 10.1007/s12630-009-9253-0 PMC7101672 20082167

[B19] Peek GJ Mugford M Tiruvoipati R Wilson A Allen E Thalanany MM Hibbert CL Truesdale A Clemens F Cooper N Firmin RK Elbourne D Efficacy and economic assessment of conventional ventilatory support versus extracorporeal membrane oxygenation for severe adult respiratory failure (CESAR): a multicentre randomised controlled trial Lancet 2009 374 9698 1351 1363 10.1016/S0140-6736(09)61069-2 19762075

[B20] Zambon M Vincent JL Mortality rates for patients with acute lung injury/ARDS have decreased over time Chest 2008 133 5 1120 1127 10.1378/chest.07-2134 18263687

[B21] Phua J Badia JR Adhikari NK Friedrich JO Fowler RA Singh JM Scales DC Stather DR Li A Jones A Gattas DJ Hallett D Tomlinson G Stewart TE Ferguson ND Has mortality from acute respiratory distress syndrome decreased over time?: A systematic review Am J Respir Crit Care Med 2009 179 3 220 227 10.1164/rccm.200805-722OC 19011152

[B22] Peris A Cianchi G Biondi S Bonizzoli M Pasquini A Bonacchi M Ciapetti M Zagli G Bacci S Lazzeri C Bernardo P Mascitelli E Sani G Gensini GF Extracorporeal life support for management of refractory cardiac or respiratory failure: initial experience in a tertiary centre Scand J Trauma Resusc Emerg Med 2010 18 1 28 10.1186/1757-7241-18-28 20487571 PMC2879235

[B23] Ventilation with lower tidal volumes as compared with traditional tidal volumes for acute lung injury and the acute respiratory distress syndrome. The Acute Respiratory Distress Syndrome Network N Engl J Med 2000 342 18 1301 1308 10.1056/NEJM200005043421801 10793162

[B24] Brogan TV Thiagarajan RR Rycus PT Bartlett RH Bratton SL Extracorporeal membrane oxygenation in adults with severe respiratory failure: a multi-center database Intensive Care Med 2009 35 12 2105 2114 10.1007/s00134-009-1661-7 19768656

[B25] Muntean W Coagulation and anticoagulation in extracorporeal membrane oxygenation Artif Organs 1999 23 11 979 983 10.1046/j.1525-1594.1999.06451.x 10564301

[B26] Herbst D Najm HK Jha KN Long-term extracorporeal circulation management: the role of low- and high-range heparin ACT tests J Extra Corpor Technol 2008 40 4 271 274 19192757 PMC4680717

[B27] Colby CE Sheehan A Benitz W Van Meurs K Halamek LP Moss RL Maintaining adequate anticoagulation on extracorporeal membrane oxygenation therapy: Hemochron Junior Low Range versus Hemochron 400 J Extra Corpor Technol 2003 35 1 35 38 12680494

[B28] Welsby IJ McDonnell E El-Moalem H Stafford-Smith M Toffaletti JG Activated clotting time systems vary in precision and bias and are not interchangeable when following heparin management protocols during cardiopulmonary bypass J Clin Monit Comput 2002 17 5 287 292 10.1023/A:1021298103264 12546261

[B29] Bouhemad B Liu ZH Arbelot C Zhang M Ferarri F Le-Guen M Girard M Lu Q Rouby JJ Ultrasound assessment of antibiotic-induced pulmonary reaeration in ventilator-associated pneumonia* Crit Care Med 2009 23 23 10.1097/CCM.0b013e3181b08cdb 19633538

[B30] Waydhas C Intrahospital transport of critically ill patients Crit Care 1999 3 5 R83 89 10.1186/cc362 11094486 PMC137237

[B31] Voigt LP Pastores SM Raoof ND Thaler HT Halpern NA Review of a large clinical series: intrahospital transport of critically ill patients: outcomes, timing, and patterns J Intensive Care Med 2009 24 2 108 115 10.1177/0885066608329946 19188270

[B32] Lidegran MK Ringertz HG Frenckner BP Linden VB Chest and abdominal CT during extracorporeal membrane oxygenation: Clinical benefits in diagnosis and treatment Acad Radiol 2005 12 3 276 285 10.1016/j.acra.2004.11.027 15766686

[B33] Jepson SL Harvey C Entwisle JJ Peek GJ Management benefits and safety of computed tomography in patients undergoing extracorporeal membrane oxygenation therapy: experience of a single centre Clin Radiol 65 11 881 886 10.1016/j.crad.2010.05.007 20933642

[B34] Linden V Palmer K Reinhard J Westman R Ehren H Granholm T Frenckner B Inter-hospital transportation of patients with severe acute respiratory failure on extracorporeal membrane oxygenation--national and international experience Intensive Care Med 2001 27 10 1643 1648 10.1007/s001340101060 11685306

[B35] Wagner K Sangolt GK Risnes I Karlsen HM Nilsen JE Strand T Stenseth LB Svennevig JL Transportation of critically ill patients on extracorporeal membrane oxygenation Perfusion 2008 23 2 101 106 10.1177/0267659108096261 18840578

[B36] Mayo PH Goltz HR Tafreshi M Doelken P Safety of ultrasound-guided thoracentesis in patients receiving mechanical ventilation Chest 2004 125 3 1059 1062 10.1378/chest.125.3.1059 15006969

